# Risk of metachronous neoplasia in early-onset colorectal cancer: meta-analysis

**DOI:** 10.1093/bjsopen/zrae092

**Published:** 2024-09-04

**Authors:** Gianluca Pellino, Giacomo Fuschillo, Rogelio González-Sarmiento, Marc Martí-Gallostra, Francesco Selvaggi, Eloy Espín-Basany, Jose Perea

**Affiliations:** Colorectal Surgery, Vall d’Hebron University Hospital, Universitat Autonoma de Barcelona UAB, Barcelona, Spain; Colorectal Surgery, Department of Advanced Medical and Surgical Sciences, Università degli Studi della Campania ‘Luigi Vanvitelli’, Naples, Italy; Molecular Medicine, Biomedical Research Institute of Salamanca (IBSAL), Salamanca, Spain; Colorectal Surgery, Vall d’Hebron University Hospital, Universitat Autonoma de Barcelona UAB, Barcelona, Spain; Colorectal Surgery, Department of Advanced Medical and Surgical Sciences, Università degli Studi della Campania ‘Luigi Vanvitelli’, Naples, Italy; Colorectal Surgery, Vall d’Hebron University Hospital, Universitat Autonoma de Barcelona UAB, Barcelona, Spain; Molecular Medicine, Biomedical Research Institute of Salamanca (IBSAL), Salamanca, Spain; Department of Surgery, Vithas Arturo Soria University Hospital, Madrid, Spain

## Abstract

**Background:**

Metachronous colorectal cancer refers to patients developing a second colorectal neoplasia diagnosed at least 6 months after the initial cancer diagnosis, excluding recurrence. The aim of this systematic review is to assess the incidence of metachronous colorectal cancer in early-onset colorectal cancer (defined as age at diagnosis of less than 50 years) and to identify risk factors.

**Methods:**

This is a systematic review and meta-analysis performed following the PRISMA statement and registered on PROSPERO. The literature search was conducted in PubMed and Embase. Only studies involving patients with early-onset colorectal cancer (less than 50 years old) providing data on metachronous colorectal cancer were included in the analysis. The primary endpoint was the risk of metachronous colorectal cancer in patients with early-onset colorectal cancer. Secondary endpoints were association with Lynch syndrome, family history and microsatellite instability.

**Results:**

Sixteen studies met the inclusion criteria. The incidence of metachronous colorectal cancer was 2.6% (95% c.i. 2.287–3.007). The risk of developing metachronous colorectal cancer in early-onset colorectal cancer *versus* non-early-onset colorectal cancer patients demonstrated an OR of 0.93 (95% c.i. 0.760–1.141). The incidence of metachronous colorectal cancer in patients with Lynch syndrome was 18.43% (95% c.i. 15.396–21.780), and in patients with family history 10.52% (95% c.i. 5.555–17.659). The proportion of metachronous colorectal cancer tumours in the microsatellite instability population was 19.7% (95% c.i. 13.583–27.2422).

**Conclusion:**

The risk of metachronous colorectal cancer in patients with early-onset colorectal cancer is comparable to those with advanced age, but it is higher in patients with Lynch syndrome, family history and microsatellite instability. This meta-analysis demonstrates the need to personalize the management of patients with early-onset colorectal cancer according to their risk factors.

## Teaser

The risk of metachronous colorectal cancers in patients with early-onset colorectal cancer is comparable to those with advanced age, but they can occur later and be associated with family history and Lynch syndrome, advocating the need to identify the ideal follow-up pathways in this patient population.

## Introduction

Colorectal cancer (CRC) incidence and mortality rates vary around the world. Globally, CRC is the third most diagnosed cancer in men and in women^1^. The geographical incidence of CRC varies over 10-fold, with the highest incidence rates in Australia and New Zealand, Europe and the USA, and the lowest rates found in Africa and South Central Asia^2^. The prognosis of CRC depends on its stage at diagnosis, and early detection increases the probability of cure^3^. Once treated with curative intent, the patient's follow-up will vary according to the stage of CRC at diagnosis, as well as the type of treatment received and the patient's risk. The National Comprehensive Cancer Network (NCCN) and the European Society for Medical Oncology (ESMO) recommend, in the general population, a colonoscopy 1 year after surgery, then 3 years later and finally, every 5 years after the last colonoscopy^3–5^.

Metachronous colorectal cancer (mCRC) refers to patients with CRC developing a second CRC neoplasia diagnosed at least 6 months after the initial cancer diagnosis^6^, excluding recurrences at the anastomotic site. Patients who have developed CRC have an increased risk of developing other lesions along the colon during follow-up. Several studies in patients with sporadic CRC have established that the frequency of mCRC is approximately 1–5%^7–9^. Nevertheless, hereditary CRC syndromes are one of the most important risk factors leading to mCRC, increasing the frequency of a second tumour in these patients by 10–20%^10,11^. Among these, the most frequent, Lynch syndrome, shows a metachronous tumour rate of 12–33% with a follow-up interval of up to 15 years, and is significantly lower in patients who underwent subtotal/total colectomy (0–6%)^12^. The risk profile can change the management options regarding surgery (more extensive surgery) and more frequent surveillance^13^.

The incidence of CRC in individuals diagnosed before the age of 50 years has been rising over the last three decades (i.e. early-onset CRC; EOCRC). The incidence of colon and rectal cancers in the USA is estimated to increase by 90.0% and 124.2% respectively, for patients aged 20 to 34 years, and by 27.7% and 46.0% respectively, for patients aged 35 to 49 years by 2030^14,15^. Although heterogeneous, this rise is also confirmed in Europe^16^. This has led to increasing research to better understand this emerging problem and how best to manage it, including enhanced surveillance. There is limited evidence regarding the management of metachronous colorectal neoplasia in this subset of patients. Only a few publications address this issue, covering the prevalence of young-onset adenoma (YOA) (9%). It demonstrates an increase with age and higher risk for metachronous advanced neoplasia after YOA diagnosis, estimated to be 6%^17^. This systematic review with meta-analysis focused on EOCRC and the risk of mCRC, as well as on factors that may be involved.

## Methods

The systematic review and meta-analysis were performed following the Preferred Reporting Items for Systematic Reviews and Meta-analysis (PRISMA)^18^ statement and registered on PROSPERO (CRD42023444057).

### Search strategy and data sources

The literature search was conducted in PubMed and Embase, with no time restrictions. The following data were extracted from included studies: first author, year of publication, country, study design, number of patients, number of patients with metachronous colorectal neoplasia, age range of patients involved and duration of follow-up. The search terms used were: ‘early onset’ or ‘young patients’ or ‘young onset’ and ‘metachronous’ and ‘colorectal cancer’. A cross-reference search was used.

### Inclusion and exclusion criteria

Only studies involving patients with EOCRC (<50 years) that provided data regarding occurrence of mCRC were included in the analysis. Studies with non-extractable data, with no full-text available and those written in languages other than English were excluded. If more than one study was conducted by the same centre, only the most recent one was included. Studies only reporting data on adenomas or neoplasms other than cancer were not considered.

### Endpoints

The primary endpoint was the risk of occurrence of mCRC in patients with EOCRC, defined as developing a second CRC neoplasia diagnosed at least 6 months after the initial cancer diagnosis, excluding recurrences at the anastomotic site, in CRC patients.

Secondary endpoints were: risk of mCRC in patients with Lynch syndrome, risk of mCRC in patients with a family history and risk of mCRC in patients with microsatellite instability (MSI).

### Statistical analysis

All collected data regarding the endpoints were reported in an Excel table showing the number of patients involved in the studies, number of mCRC observed, number of patients with Lynch syndrome, MSI and family history of CRC.

A proportional meta-analysis was performed to assess the mean rate of mCRC using MedCalc® statistical software. Statistical heterogeneity was assessed using *P* values of the Cochrane Q test and inconsistency (*I^2^*) statistics.

The comparative meta-analysis on risk in younger *versus* older patients was performed with the same software, and the estimated effect measures are reported as odds ratios (OR) with 95% confidence intervals (c.i.).

### Risk-of-bias assessment and quality of the studies

The Joanna Briggs Institute's (JBI) critical appraisal checklist for studies reporting prevalence data was utilized to evaluate the quality of the studies^19^. This tool assessed studies according to 10 questions. If the answer was yes, the question was assigned a score of 1. If the answer was no, unclear or not applicable, a score of 0 was assigned.

## Results

**Fig. 1:**
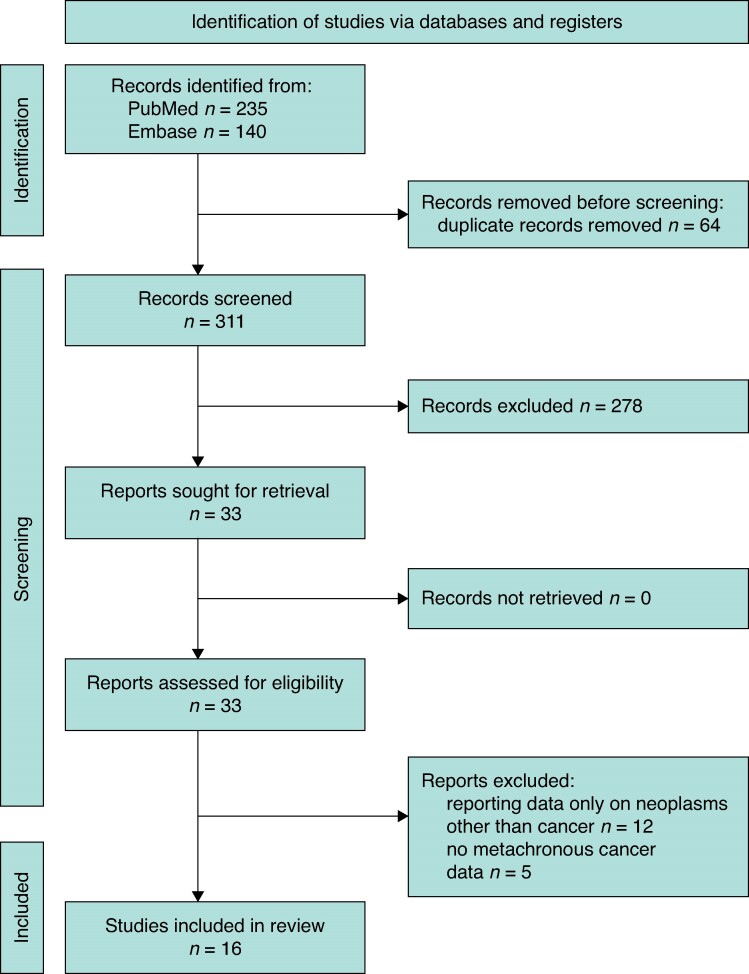
**PRISMA flow chart**Flow chart of study selection for the present meta-analysis according to the PRISMA statement.

The search yielded 375 studies, 235 from MEDLINE (PubMed) and 140 from Embase. The criteria used for the search were (‘early onset’ or ‘young patients’ or ‘young onset’) and ‘metachronous’ and ‘colorectal cancer’. The search was performed on 31 October 2023. Titles and abstracts were assessed; 71 duplicates were excluded. Reviews, meta-analyses and case reports were excluded. Full-text articles were reviewed, with eight being excluded owing to lack of relevant data. Sixteen studies met the inclusion criteria^20–35^. There was 100% agreement among reviewers in the extraction of data, reported in a Microsoft® Excel spreadsheet. The PRISMA flow chart selection is shown in *Fig. 1*.

In total, 7865 patients with CRC were included in the analysis. The characteristics of the included studies are summarized in *Table 1*.

**Table 1:** Characteristics of the included studies

Author	Year	Country	Study design	Mean follow-up (days)	Definition of early onset	Mean age (years)	No. of patients	Risk-of-bias assessment
Myers *et al.*^30^	2013	USA	Retrospective	NA	<50	41.4	180	7
Chen *et al.*^21^	2021	USA	Retrospective	45	18–49	43	107	8
Kim *et al.*^25^	2016	Korea	Retrospective	66.4	<45	38	693	9
Parry *et al.*^31^	2011	New Zealand	Retrospective	96	<49	NA	222	8
Aronson *et al.*^20^	2015	Canada	Retrospective	74.4	<35	29.7	285	9
Djursby *et al.*^22^	2020	Denmark	Prospective	NA	<40	36	50	10
Ikenaga *et al.*^23^	2002	Japan	Prospective	NA	<40	34.7	53	8
Kozak *et al.*^28^	2017	USA	Retrospective	68	<50	33.9	150	10
Kim *et al.*^26^	2017	Korea	Retrospective	NA	<50	43.7	95	9
Win *et al.*^35^	2013	Australia	Retrospective	132	<50	42.8	79	8
Samadder *et al.*^32^	2014	USA	Retrospective	NA	<50	43.2	1749	9
Klos *et al.*^27^	2014	USA	Retrospective	24	<50	NA	301	8
Tian *et al.*^33^	2020	China	Retrospective	54.9	<50	NA	74	10
Tjaden *et al.*^34^	2021	USA	Retrospective	NA	<50	NA	92	8
Lee *et al.*^29^	2016	Korea	Retrospective	NA	<50	NA	3205	10
Kim *et al.*^24^	2017	Korea	Retrospective	36	<50	NA	530	9

NA, not available.

### mCRC in patients with EOCRC

**Fig. 2:**
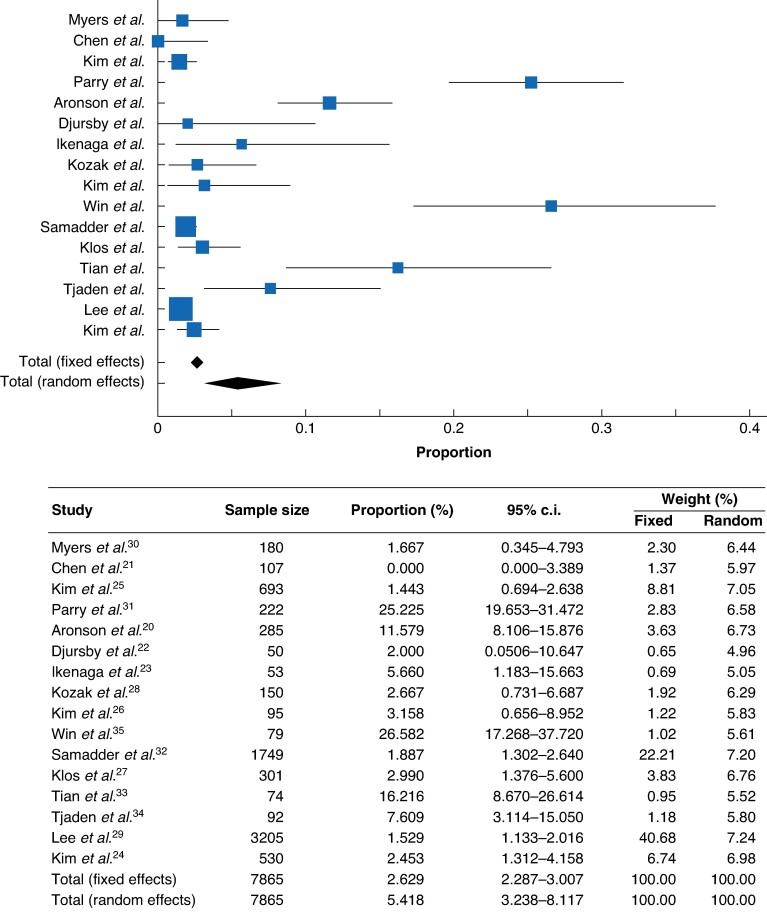
**Proportion meta-analysis of metachronous colorectal cancer (mCRC) in early-onset colorectal cancer (EOCRC)**Forest plot showing the rates of mCRC in EOCRC among the included studies.

All included studies provided data regarding number of mCRC in young patients; 7865 patients under 50 years with CRC history were included. Among them, 257 developed mCRC. The rate of mCRC in patients with CRC was 2.6% (95% c.i. 2.287 to 3.007, *I^2^* 94.38%, *P* < 0.001) as shown in *Fig. 2*.

**Fig. 3:**
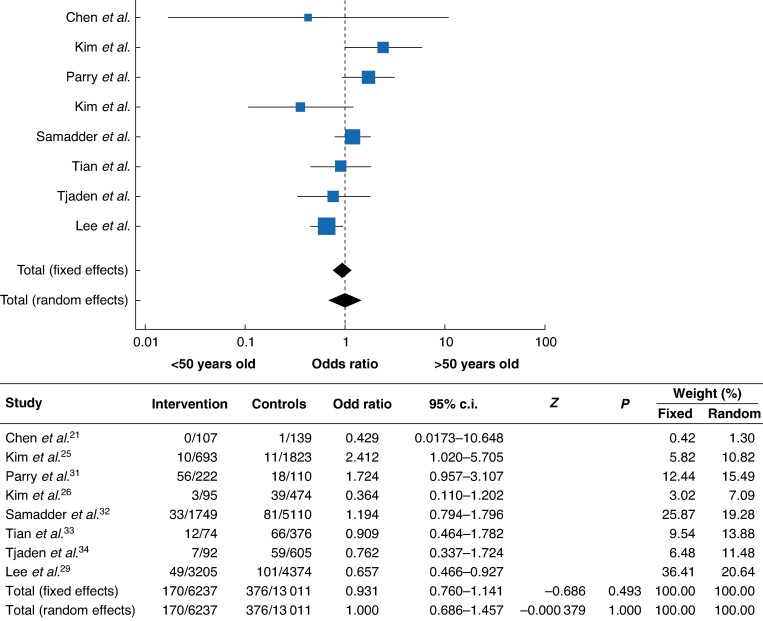
**Meta-analysis of metachronous colorectal cancer (mCRC) in young *versus* old population**mCRC in young (<50 years) *versus* older (>50 years) patients. Forest plot with odds ratio of single studies and overall odds ratio.

The risk of developing mCRC in young *versus* older patients was assessed (*Fig. 3*). Eight studies reported data on patient over 50 years; the meta-analysis showed: OR 0.93, 95% c.i. 0.760 to 1.141, *I^2^* 59.07%, *P* = 0.017.

### Risk of mCRC in patients with Lynch syndrome under the age of 50 years

**Fig. 4:**
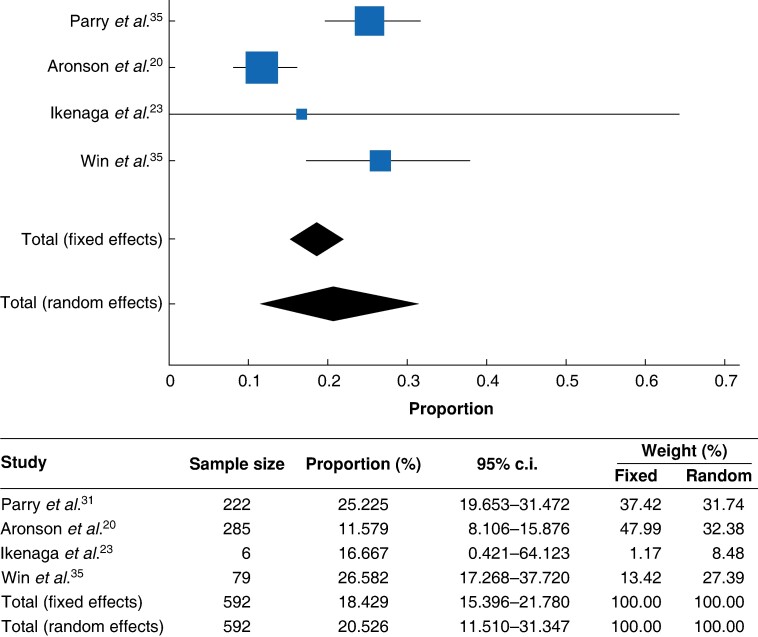
**Proportion meta-analysis of metachronous colorectal cancer (mCRC) in early-onset colorectal cancer (EOCRC) in patients with Lynch syndrome**A forest plot showing the rates of mCRC in EOCRC in patients with Lynch syndrome among the included studies.

Four studies^20,23,31,35^ reported data on patients with Lynch syndrome. Of 592 patients with EOCRC and Lynch syndrome, 111 developed mCRC (18.43%, 95% c.i. 15.396 to 21.780, *I^2^* 84.82%, *P* < 0.001). The proportional meta-analysis is reported in *Fig. 4*.

### Risk of mCRC in patients with family history under the age of 50 years

*Fig. S1* shows the proportional meta-analysis regarding the association of family history with a rate of 10.52% (95% c.i. 5.555 to 17.659, *I^2^* 81.20%, *P* = 0.005).

### Risk of mCRC in patients with MSI under the age of 50 years

Two studies provided data on correlation with MSI. The proportion of mCRC tumours in an MSI patient population with EOCRC was 19.7% (95% c.i. 13.583 to 27.086, *I^2^* 26.90%, *P* = 0.242, *Fig. S2*).

### Quality of studies

The overall strength of evidence is summarized in *Table S1*. Low risk of bias was found for 94% of the studies, scoring between 8 and 10. One study had a medium risk (score = 7).

## Discussion

This systematic review with meta-analysis analysed mCRC in patients with EOCRC. The percentage of mCRC in EOCRC was found to be 2.6% compared with 2% reported in the literature with no age restriction^36^. The present meta-analysis focused specifically on the age of onset of CRC and demonstrated no increased risk of mCRC in EOCRC compared with the older population. This finding was also confirmed by another recent meta-analysis that demonstrated a pooled risk ratio per year of 1.05, 95% c.i. 0.96 to 1.14^37^.

An important factor to consider in this population is the association with other risk factors for mCRC such as Lynch syndrome. Germline pathogenic variants in known cancer predisposition genes appear in approximately 13% (range 9–26%) of patients with EOCRC^38^.

In the present study, this association increases the rate of mCRC to 18.43% in patients with EOCRC. This might justify a more extensive resection of the first tumour in young patients with CRC and Lynch syndrome. In fact, a recent meta-analysis on the risk of mCRC in patients with Lynch syndrome showed a lower risk rate (6% *versus* 22.8%) in patients undergoing extensive compared with segmental resection^13^. It could be proposed that the younger the patient, the higher the risk of mCRC, and the potential benefit from extended resection of the first tumour. In patients with Lynch syndrome, the management should be personalized, as the risk of mCRC after partial colectomy in carriers of low-risk variants has shown to be similar to that reported after extensive colectomy in carriers of high-risk variants, suggesting that non-extensive colectomies together with an endoscopic surveillance are sufficient in carriers of low-risk Lynch syndrome variants^39,40^.

Despite the importance of lifelong follow-up, the risk is not only higher with Lynch syndrome in younger individuals with CRC, but also for metachronous advanced neoplasia^40^. Patients with a family history of CRC also need to be followed up more rigorously and with shorter intervals. The present review found a 10.52% rate of mCRC in EOCRC patients with a family history of CRC, which suggests that such patients also need careful attention. A family history of CRC is known to be associated with an increased risk of metachronous neoplasia^41^.

Surveillance strategies after EOCRC treatment are not well defined, except for patients with a known hereditary CRC syndrome. A previous study^42^ demonstrated that 47% of EOCRC patients did not develop polyps after surgical treatment (with a follow-up longer than 7 years). Surveillance strategies need to be clarified and stratify patients with EOCRC to define correct intervals and individualized management. In general, the recommendation following a CRC resection is an initial follow-up colonoscopy after 1 year to identify any metachronous neoplasia; if negative, the next endoscopy is recommended at 3 years. Shorter intervals are advised based on the patient's age, family history and hereditary non-polyposis CRC status^43^. These patients need long follow-up given their young age, irrespective of the genetic and/or familial predisposition. Studies on EOCRC with longer intervals of surveillance are needed to define the appropriate approach. Patients with EOCRC should be made aware that they will need to be followed up for a longer time, until more evidence is produced.

MSI is also known to be associated with increased risk of mCRC, mostly due to patients with Lynch syndrome. Shitoh *et al.* demonstrated an almost six-fold increased risk in patients with MSI (OR 5.83, 95% c.i. 2.06 to 16.42)^44^. The current review showed that the proportion of mCRC tumours in the MSI patient population with EOCRC was 19.7%. Ikenaga *et al.* demonstrated that the frequency of MSI in patients with CRC is significantly higher than in the elderly population (50.1 *versus* 12–21% respectively). The authors conclude that microsatellite analysis and family history interviews are useful tools for the assessment of these patients^23^. Therefore, it is crucial, even more so in young patients, to have a preoperative diagnosis of Lynch syndrome or MSI to guide the best surgical treatment for these patients. The American College of Gastroenterology guidelines recommend, in all newly diagnosed CRCs, to evaluate patients for mismatch repair deficiency^45^.

It is also important to emphasize the importance of somatic testing on endoscopic biopsy and immunohistochemistry for Mismatch Repair (MMR) proteins or MSI testing. The challenge is timely access to rapid testing for Lynch syndrome, as patients on an oncology pathway need rapid treatment. In England, the National Institute of Health and Care Excellence (NICE) has developed national guidelines recommending universal testing for Lynch syndrome in people with colorectal (CRC, DG27) and endometrial (EC, DG42) cancer^46^.

There are three somatic investigations that can be used to detect MMR deficiency. Immunohistochemical analysis (IHC) identifies abnormally formed MMR proteins and may indicate the underlying gene in which MMR deficiency is present, or alternatively, PCR-based MSI testing. These tests can be performed as index tests in patients with CRC to identify those who might benefit from further evaluation for Lynch syndrome.

The findings of the present review should be interpreted with caution, as studies used for the meta-analysis were retrospective, and a selection bias could not be excluded. Heterogeneity of the data was moderate or high in some analyses. However, most studies had a low risk of bias, with only one found to have intermediate bias. The quantitative analysis of data obtained from studies performed on patient populations from different countries makes it possible to generalize the practical findings at a global level.

## Supplementary Material

zrae092_Supplementary_Data

## Data Availability

Additional data can be found as supplementary material and are available from the corresponding author on reasonable request.
