# Supplementary material for: Risk of metachronous neoplasia in early-onset colorectal cancer: meta-analysis
Source: BJS Open. 2024 Sep 4;8(5):zrae092. doi: 10.1093/bjsopen/zrae092 (PMC11373379; doi:10.1093/bjsopen/zrae092)
Supplement: zrae092_Supplementary_Data [file zrae092_supplementary_data.zip › Supplementary_material.docx]

**Risk of metachronous neoplasia in early onset colorectal cancer: Systematic review and meta-analysis**

Gianluca **Pellino**^1^*, Giacomo **Fuschillo**^2^*, Rogelio **González-Sarmiento**^3^, Marc **Martí Gallostra**^1^, Francesco **Selvaggi**^2^, Eloy **Espín-Basany**^1^, Jose **Perea**^3, 4^

*1 Colorectal surgery, Vall d’Hebron University Hospital, Universitat Autonoma de Barcelona UAB, Barcelona, Spain*

*2 Colorectal surgery, Department of Advanced Medical and Surgical Sciences, Università degli Studi della Campania “Luigi Vanvitelli”, Naples, Italy*

*3 Biomedical Research Institute of Salamanca (IBSAL), Salamanca, Spain*

*4 Department of Surgery. Vithas Arturo Soria University Hospital, Madrid, Spain.*

** G Pellino and G Fuschillo equally contributed to this manuscript and share the first author position*

**Correspondence to:**

- Gianluca Pellino, MD, PhD, FRCS, FRCP, FEBS (Coloproctology), FACS, Colorectal surgery, Vall d’Hebron University Hospital, Universitat Autonoma de Barcelona UAB, Passeig de la Vall d’Hebron 119-129, 08035, Barcelona, Spain. Email: [gianluca.pellino@uab.cat](mailto:gianluca.pellino@uab.cat) [gipe1984@gmail.com](mailto:gipe1984@gmail.com)
- Jose Perea, MD, PhD, MSc, Biomedical Research Institute of Salamanca (IBSAL), Salamanca, Spain; Department of Surgery. Vithas Arturo Soria University Hospital, Madrid, Spain. Electronic address: [josepereag@hotmail.com](mailto:josepereag@hotmail.com)

**Supplementary Materials - Index**

| **Supplementary Figures and Tables** |  |
| --- | --- |
| Supplementary Figure 1 | *pag. 2* |
| Supplementary Figure 2 | *pag. 3* |
| Supplementary Table 1 | *pag. 4* |

**Supplementary Figure 1**

**
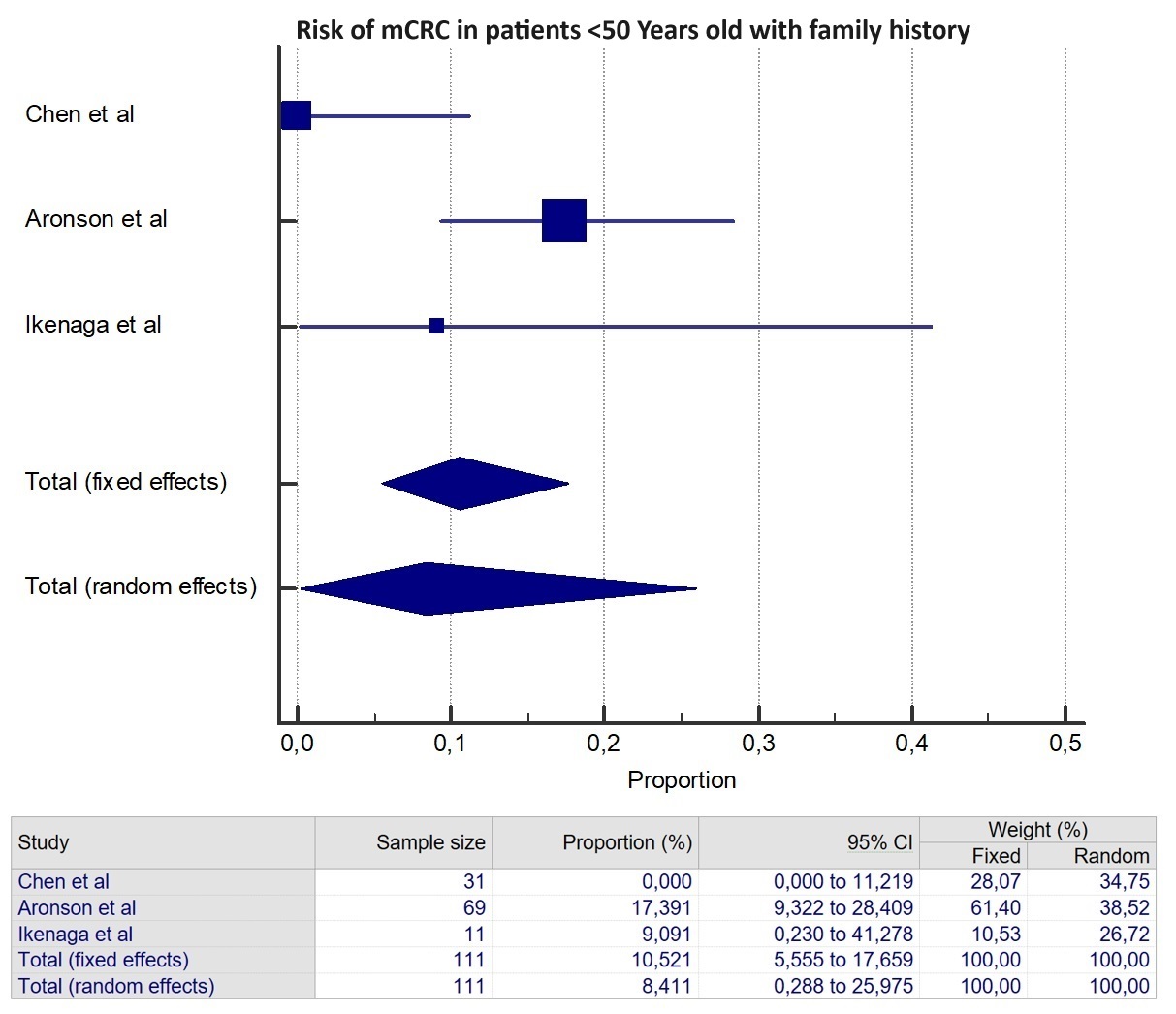
**

**Supplementary Figure 2**

**
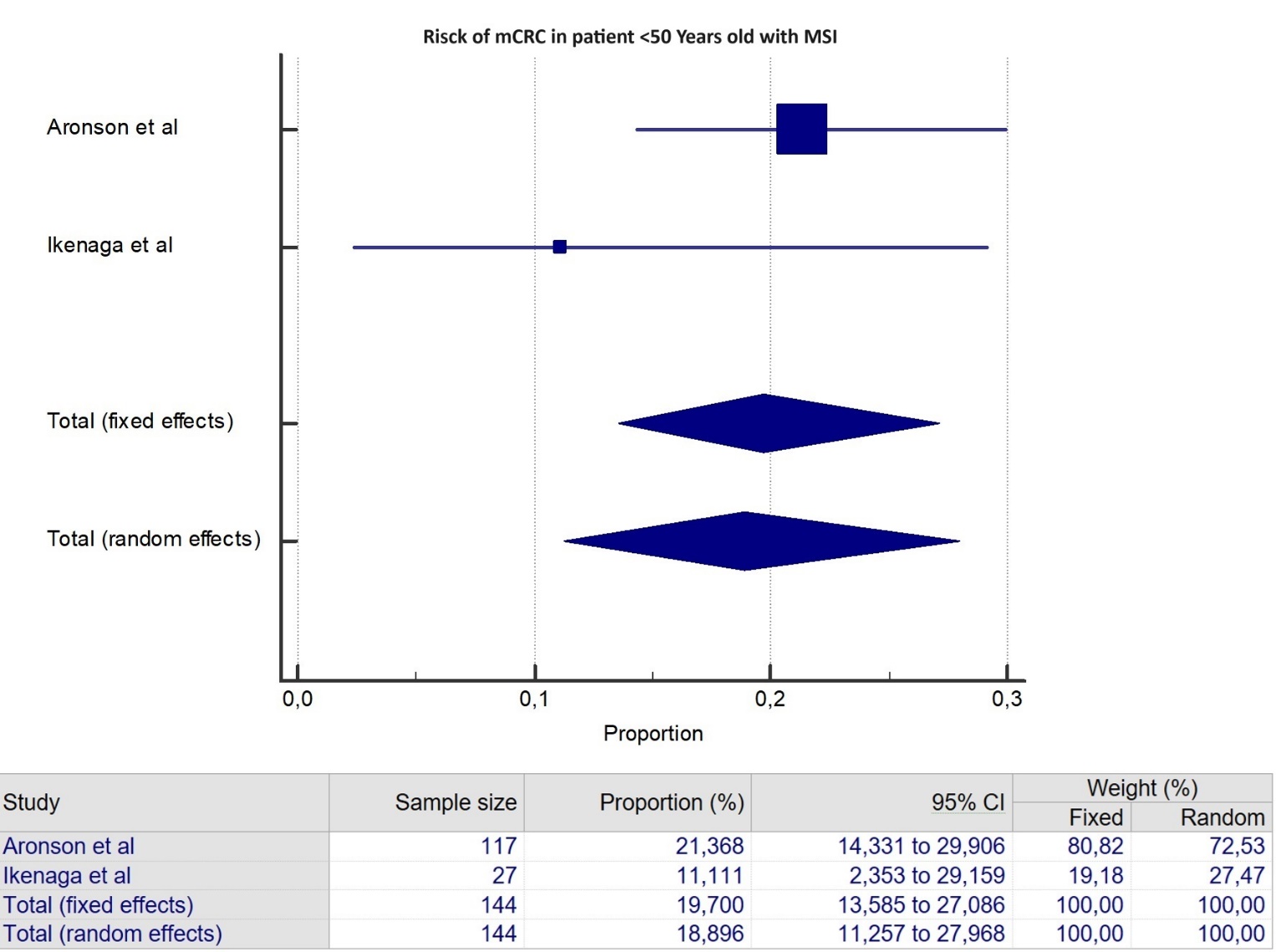
**

**Supplementary Table 1**

| **CASE SERIES** | | | | | | | | | | | |
| --- | --- | --- | --- | --- | --- | --- | --- | --- | --- | --- | --- |
| **Articles** | **Q1** | **Q2** | **Q3** | **Q4** | **Q5** | **Q6** | **Q7** | **Q8** | **Q9** | **Q10** | **SCORE** |
| Myers et al | Y | Y | Y | Y | U | N | Y | Y | N | Y | 7 |
| Chen et al | Y | Y | Y | Y | Y | N | Y | Y | N | Y | 8 |
| Kim J et al | Y | Y | Y | Y | U | Y | Y | Y | Y | Y | 9 |
| Parry et al | Y | Y | Y | U | U | Y | Y | Y | Y | Y | 8 |
| Aronson et al | Y | Y | Y | U | Y | Y | Y | Y | Y | Y | 9 |
| Djursby et al | Y | Y | Y | Y | Y | Y | Y | Y | Y | Y | 10 |
| Ikenaga et al | Y | Y | Y | Y | Y | N | Y | Y | N | Y | 8 |
| Kozak et al | Y | Y | Y | Y | Y | Y | Y | Y | Y | Y | 10 |
| Kim S et al | Y | Y | Y | U | Y | Y | Y | Y | Y | Y | 9 |
| Win et al | Y | Y | Y | Y | Y | U | Y | Y | U | Y | 8 |
| Samadder et al | Y | Y | Y | Y | U | Y | Y | Y | Y | Y | 9 |
| Klos et al | Y | Y | Y | Y | U | Y | N | Y | Y | Y | 8 |
| Tian et al | Y | Y | Y | Y | Y | Y | Y | Y | Y | Y | 10 |
| Tjaden et al | Y | Y | Y | U | U | Y | Y | Y | Y | Y | 8 |
| Lee Y et al | Y | Y | Y | Y | Y | Y | Y | Y | Y | Y | 10 |
| Kim H et al | Y | Y | Y | Y | U | Y | Y | Y | Y | Y | 9 |
